# Crack Control in Additive Manufacturing by Leveraging Process Parameters and Lattice Design

**DOI:** 10.3390/mi15111361

**Published:** 2024-11-10

**Authors:** Jun Hak Lee, Seong Je Park, Jeongho Yang, Seung Ki Moon, Jiyong Park

**Affiliations:** 1 Advanced Joining & Additive Manufacturing R&D Department, Korea Institute of Industrial Technology, 156 Gaetbeol-ro, Yeonsu-Gu, Incheon 21999, Republic of Korea; junhak.lee@lignex1.com (J.H.L.); junghotoo@kitech.re.kr (J.Y.); 2 C5ISR Mechanical R&D Laboratory, LIG Nex1, 333 Pangyo-ro, Bundang-gu, Seongnam-si 13488, Gyeonggi-do, Republic of Korea; 3 Singapore Centre for 3D Printing, School of Mechanical and Aerospace Engineering, Nanyang Technological University, 50 Nanyang Avenue, Singapore 639798, Singapore; seongje.park@ntu.edu.sg (S.J.P.); skmoon@ntu.edu.sg (S.K.M.); 4 Department of Convergence Manufacturing System Engineering, University of Science and Technology (UST), 217 Gajeong-ro, Yuseong-gu, Daejeon 34113, Republic of Korea

**Keywords:** additive manufacturing, crack propagation, lattice structures, impact energy

## Abstract

This study investigates the design of additive manufacturing for controlled crack propagation using process parameters and lattice structures. We examine two lattice types—octet-truss (OT) and diamond (DM)—fabricated via powder bed fusion with Ti-6Al-4V. Lattice structures are designed with varying densities (10%, 30%, and 50%) and process using two different laser energies. Using additive-manufactured specimens, Charpy impact tests are conducted to evaluate the fracture behavior and impact energy levels of the specimens. Results show that the type of the lattice structures, the density of the lattice structures, and laser energy significantly influence crack propagation patterns and impact energy. OT exhibits straighter crack paths, while DM demonstrates more random fracture patterns. Higher-density lattices and increased laser energy generally improve the impact energy. DM consistently outperformed OT in the impact energy for angle specimens, while OT showed superior performance in stair specimens. Finally, a case study demonstrates the potential for combining OT and DM structures to guide crack propagation along predetermined paths, offering a novel approach to protect critical components during product failure.

## 1. Introduction

Additive manufacturing (AM) has emerged as a transformative technology that enables the production of complex geometries and the customization of material properties through layer-by-layer manufacturing [1,2,3,4]. Based on the materials and mechanism of deposition, AM technologies are divided into seven groups according to ISO/ASTM 52900 as follows: binder jetting, directed energy deposition, material extrusion, material jetting, powder bed fusion (PBF), sheet lamination, and vat photopolymerization (VPP) [5,6]. Among them, PBF is actively used due to its advantage of being able to produce precision metal products with micro levels such as lattice structures and minimal surfaces that cannot be fabricated using traditional technologies [7,8,9,10].

Most researchers studied lattice structures fabricated by AM for verifying mechanical properties, enhancing thermal exchange, structural optimization, and being lightweight. Pham et al. researched the comprehensive physical behavior of lattice structures inspired by crystal microstructure [11]. They introduced the design with desired properties by combining the hardening principles of metallurgy and architected materials. Park et al. studied one of the methods to enhance the mechanical properties of lattice structures via hybrid lattice structures [12]. The authors found that stacking two lattice structures to fabricate a part improved energy absorption. In terms of thermal exchange, Zhou et al. experimentally and numerically investigated the heat performance of various lattice structures. They claimed that thermal conductivity can increase up to 50.67% by enlarging the minimum cross-section area with the main heat conduction direction under the same porosity and specific surface area [13]. Kang et al., Huang et al., and Maskery et al. focused on both structural optimization and weight. They investigated the structural optimization of various lattice structures according to Maxwell’s stability criterion. The authors applied lightweight lattice structures to various industrial fields [14,15,16].

Based on the previous research, the authors of this study adopted lattice structures as a path for crack propagation having characteristics of relatively lower mechanical properties and lighter than solids. With a similar idea, there is research that uses VPP to fabricate lattice structures and induce cracks [17]. These findings not only validate the use of lattice structures to control crack propagation but also demonstrate their practical application in real situations. However, there is a clear difference in terms of materials, types of lattice structures, AM method, and external force applied to the specimens compared to this study.

Thus, this study investigates crack propagation in lattice structures, octet-truss (OT), and diamond (DM), fabricated via PBF using Ti-6Al-4V, a material known for its high strength and corrosion resistance. By fabricating the impact specimen using various densities of lattice structures (10%, 30%, and 50%) and two different laser energies, we aim to systematically evaluate how these variables affect the crack propagation pattern and impact energy. Furthermore, using the specimens with angles and stairs having various geometry, crack propagation and the impact energy of OT and DM are verified according to two laser energies. The proposed method can contribute to extending their functional life with optimized crack propagation by effectively managing the failure modes.

## 2. Experimental

### 2.1. PBF AM Process

Utilizing a commercially accessible PBF system (DMP flex 350, 3D systems, Rock Hill, SC, USA) and Ti-6Al-4V (LaserForm Ti Gr23, 3D Systems, Rock Hill, SC, USA) powder with an average particle size of 33 μm, the lattice structures were fabricated in an argon atmosphere to prevent oxidation of Ti-6Al-4V (Oxygen concentration: approximately 3 ppm). The laser power, scan speed, layer thickness, and hatching distance were performed according to the manufacturer`s recommended two parameter sets of 145 W, 1000 mm/s, 30 μm, and 82 μm for high energy and of 125 W, 2800 mm/s, 30 μm, and 110 μm for low energy, respectively. Among various mechanical properties of Ti-6Al-4V, AMed Ti-6Al-4V has a Charpy impact energy of 10.86 J for high laser energy and 2.70 J for low laser energy, respectively, according to our experimental results.

### 2.2. Selection of Lattice Structures

**Figure 1:**
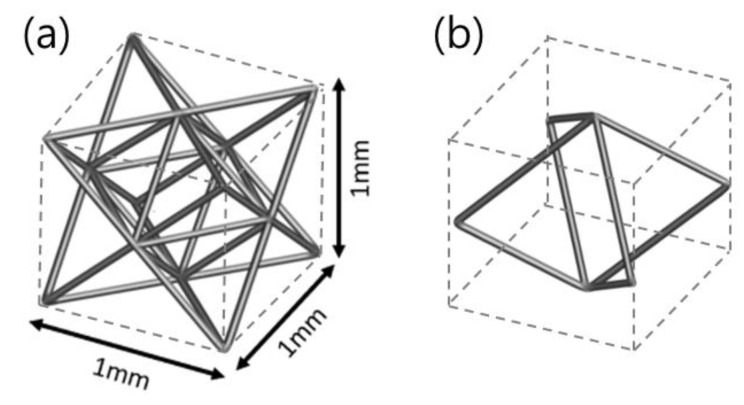
The lattice structure for (**a**) OT and (**b**) DM with unit cell size.

As shown in Figure 1, OT and DM were selected according to Maxwell’s stability criterion as stretch- and bending-dominant structures, respectively. The stretch-dominant structures have high structural efficiency and bending-dominant structures have superior energy absorption [14,18]. OT has several struts of 36 and several joints of 14. DM has some struts of 8 and some joints of 6, respectively. OT exhibits a slightly more complex strut alignment than DM. OT and DM had 1 × 1 × 1 mm unit cell sizes with densities of 10, 30, and 50%. The density was changed by increasing the thickness of the struts while fixing the unit cell size. All lattice structures were designed by 3DXpert V23 (3D systems, Rock Hill, SC, USA) software.

### 2.3. Charpy Impact Tests

**Figure 2:**
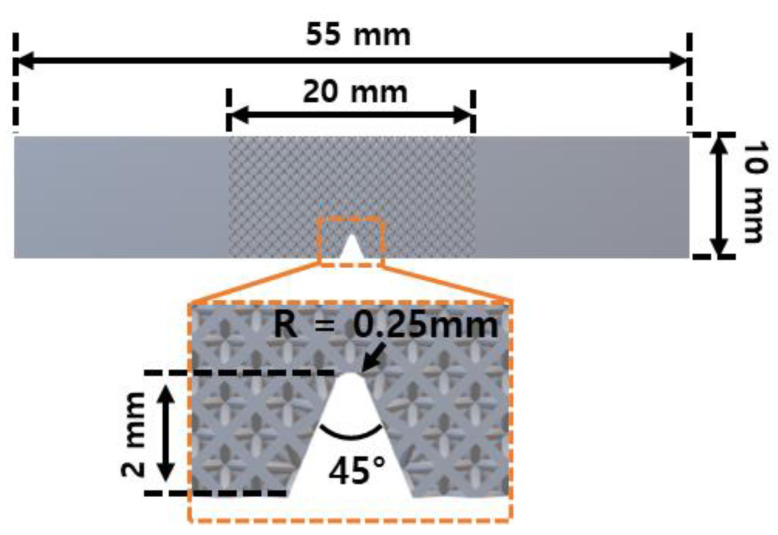
Dimensions of Charpy impact specimens with lattice structures.

The impact specimens were fabricated according to ASTM E23: standard test methods for notched bar impact testing of metallic materials [19,20]. The specimen used in this study was manufactured with lattice structures having a length of 20 mm inside the Charpy impact specimen. The V-notch was manufactured simultaneously with the Charpy impact specimens by AMed processing without separate post-processing. The geometry information of the Charpy impact specimen is shown in Figure 2. The impact test machine (IT 406, Tinius Olsen, UK) used in the experiment has a measurement capacity of 406 J, a drop height of 1.52 m, and an impact velocity of 5.47 m/s. All Charpy impact tests were conducted at room temperature. Each test was carried out using three specimens fabricated, thus showing value with standard deviation.

### 2.4. Design of Specimens for Crack Guidance

**Figure 3:**
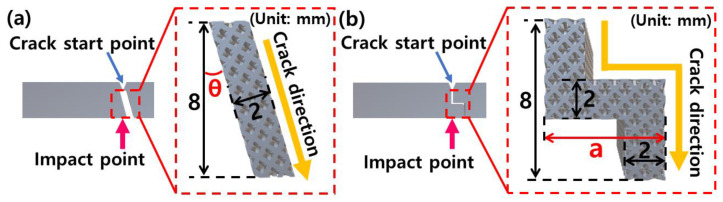
Geometry information of specimens for crack guidance: (**a**) angle and (**b**) stair.

The specimens for crack guidance were designed to derive the optimal conditions for inducing cracks according to the types of lattice structures, the density of lattice structures, and laser energy. Two types of crack guidance specimens such as angle and stair were designed as shown in Figure 3. Impact tests were conducted while increasing θ (Figure 3a) and a (Figure 3b). It was set to 15–60°, and 5–10 mm for the θ and a, respectively. Crack induction and impact strength for each specimen were measured according to the types of lattice structures, the density of lattice structures, and laser energy.

## 3. Results and Discussions

### 3.1. Physical Properties of the AMed Specimens with Lattice Structures

**Figure 4:**
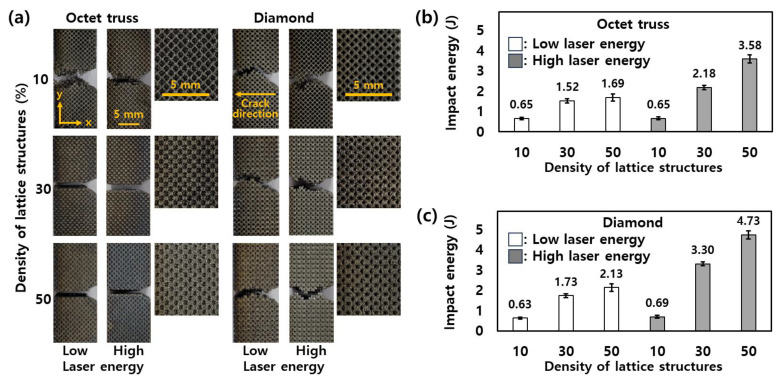
(**a**) Fracture pattern according to the lattice structures and laser energies. The impact energy of (**b**) OT and (**c**) DM according to the laser energies and density of lattice structures.

The fracture pattern of the lattice structure after an impact test is shown in Figure 4a. OT and DM show completely different fracture patterns at densities above 30%, but more similar patterns at a density of 10%. In the case of OT, the fracture pattern is a straight line parallel to the impact direction. In contrast, for DM, cracks propagate randomly as soon as DM breaks. As the density of the lattice structures increases, the empty space inside the lattice structures decreases. Accordingly, the strut diameter of DM becomes larger than that of OT because OT has more struts than DM inside the unit cell as shown in Figure 1. That is, the struts of OT are packed more densely than the DM in one unit cell. The dense arrangement of struts makes it easy for fracture to propagate to the adjacent struts when impacted, resulting in a straight fracture pattern. In contrast, DM, which has a small number of struts in a unit cell and thus a large space, shows an irregular fracture pattern when impacted, because it is difficult for the impact to propagate to the adjacent strut [18,21]. From this perspective, OT with a low density of 10% and relatively far strut-to-strut distance exhibits irregular fracture patterns like DM. The fracture pattern was observed to be lattice-dependent, regardless of the laser energy.

Figure 4b,c show the impact energy of OT and DM according to the laser energies and density of lattice structures, respectively. Both OT and DM show low impact energies of less than 0.7 J when the density of lattice structures is 10%. Additionally, DM has higher impact energy than OT at the same density. This demonstrates that DM structures with fewer but thicker struts in the unit cell are better suited to absorb and dissipate impact energy [12,16]. The larger strut diameter in DM, especially at higher densities, likely contributes to this increased capacity for energy absorption [22]. Furthermore, the irregular crack propagation pattern observed in DM, as mentioned earlier, may also play a role in delaying complete failure, allowing the structure to absorb more energy before breaking. Interestingly, the difference between low and high laser energy is more evident at higher densities of lattice structures for both OT and DM. At 50% density, for instance, high laser energy leads to a considerable improvement in impact energy, particularly in OT and DM, where the impact energy increases from 2.18 J to 3.58 J and from 3.3 J to 4.73 J, respectively. This suggests that higher laser energy facilitates the formation of more robust lattice structures, enhancing their ability to withstand impact. Thus, the DM at high laser energy shows superior energy absorption capabilities compared to OT, making it more suitable for applications requiring higher impact resistance.

### 3.2. Crack Behavior of AMed Specimens with Lattice Structures

**Figure 5:**
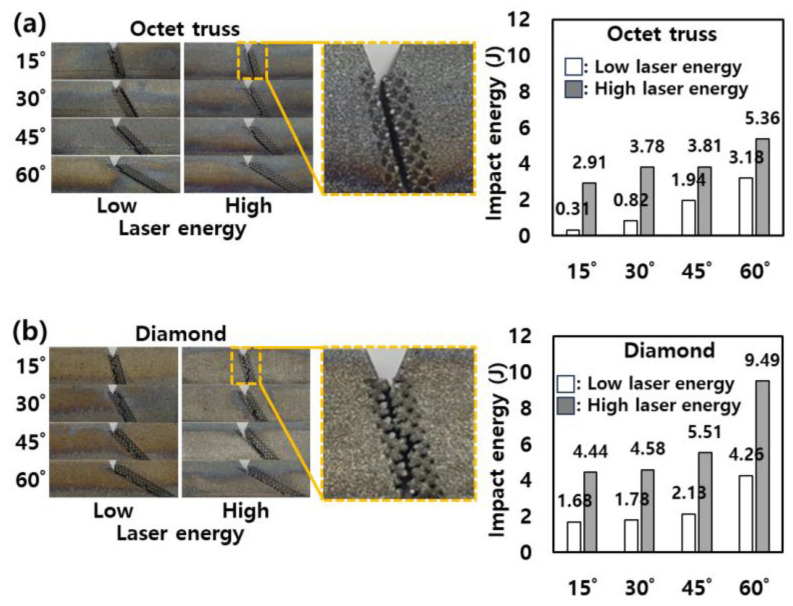
Crack propagation pattern and impact energy of angle specimens with (**a**) OT and (**b**) DM in two laser energies.

In Section 3.1, a comprehensive evaluation of lattice structures with varying densities was conducted to determine the optimal conditions that result in the highest impact energy (50% density of OT and DM). The conditions exhibiting the maximum impact energy at both laser energy levels were subjected to crack propagation analysis. The results of these tests are depicted in Figure 5, which presents the crack propagation patterns and the corresponding impact energy values for angle specimens with different angles, laser energies, and lattice structure types. The images in Figure 5 clearly show that, regardless of the specimen angle, crack propagation consistently followed the path along the lattice structure for both OT and DM configurations. When comparing the performance of the two lattice types, it becomes evident that the DM lattice consistently exhibited higher impact energy absorption compared to the OT lattice under identical conditions. This trend was observed across all tested angles, suggesting a robust correlation between types of lattice structures and mechanical performance. The superior impact resistance of the DM lattice can be attributed to its thicker struts, which provide greater structural integrity and resistance to deformation during impact [12]. Thicker struts in the DM structure led to an increased load-bearing capacity, effectively dispersing the energy from impact more efficiently than the thinner struts observed in the OT structure [23]. This result aligns closely with the findings from Section 3.1, where the impact energy values were directly proportional to the strut thickness.

**Figure 6:**
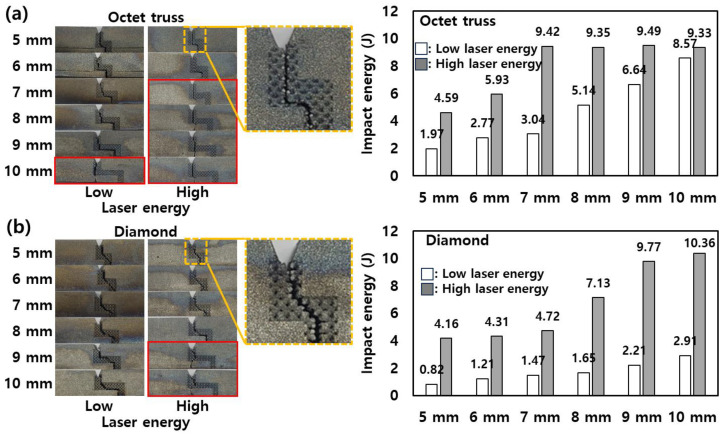
Crack propagation pattern and impact energy of stair specimens with (**a**) OT and (**b**) DM in two laser energies. (Red box: cases in which a crack does not propagate along the lattice structures.).

Figure 6 presents the crack propagation patterns and the corresponding impact energy values for stair-shaped specimens, which were fabricated under varying conditions of stair length, laser energy levels, and lattice structure types. A comparison of these results with the angle specimens shown in Figure 5 reveals significant differences in crack behavior between the angle and stair. In contrast to the angle specimens, where cracks uniformly propagated along the lattice structures regardless of the angle, the stair specimens exhibited more complex fracture behavior. Specifically, some cracks did not propagate through the lattice structures, as highlighted by the red boxes in Figure 6. This deviation in crack behavior suggests that the interaction between the lattice structure and the geometry of the stair specimens plays a pivotal role in determining the fracture path. Particularly in Figure 6b, it can be observed that the conditions under which crack propagation consistently followed the lattice structure across all stair lengths were limited to specimens with DM fabricated using low laser energy. This finding indicates that lower laser energy provides a more conducive environment for crack propagation along the predefined lattice paths in the DM. For the specimens where the cracks did not follow the lattice structure, a notable increase in impact energy was observed. These cases exhibited impact energy values in the range of 8.57 to 10.36 J for both high and low laser energy, which is significantly higher than the impact energies recorded for cracks propagating along the lattice structure. This increase in impact energy can be attributed to the additional fracture of the solid sections of the stair specimens. The propagation of cracks outside the lattice structure engages the solid parts in the fracture process, which demands more energy to initiate and propagate the crack [24].

Interestingly, the trend of impact energy in the stair specimens deviates from the behavior observed in the angle specimens. While the DM generally displayed higher impact energy in the angle specimens, the stair specimens exhibit an inverse relationship, where the OT outperforms the DM in terms of impact energy. As shown in Figure 4, the propagation of OT had a straighter line than DM, which had random propagation. This characteristic is prominent in the stair specimens. For OT, the fracture shows a straight line along the stair shape as shown in Figure 6a. However, DM tends to propagate cracks over the minimum distance due to its random fracture characteristics rather than following a stair as shown in Figure 6b. Thus, this difference in crack propagation behavior directly contributes to the observed impact energy values. The longer crack path in the OT specimen requires more energy to fully fracture the structure, thus leading to higher impact energy absorption [25]. Conversely, the shorter crack path in the DM reduces the energy required for fracture, resulting in lower impact energy values for the stair specimen compared to the OT.

### 3.3. Case Study

**Figure 7:**
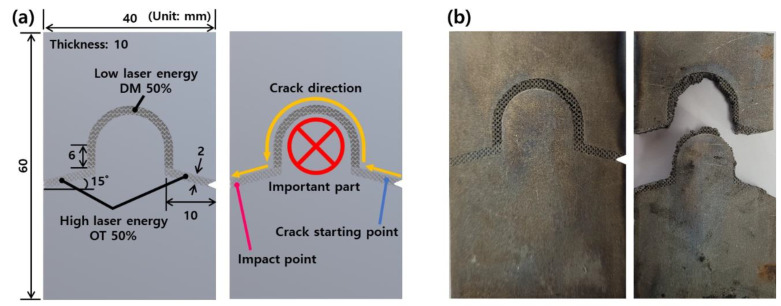
(**a**) Design of a part for a case study. (**b**) Before and after impact test of designed part.

To apply to various industries in the future, this section conducted a crack propagation experiment through a case study. The objective of this experiment is to explore how crack propagation can be controlled through the selective application of OT and DM. As shown in Figure 7a, the dimensions are set and it is assumed that there is an important part in the center. In other words, it is designed to avoid the important part when the product breaks for recycling of the important part. As a result of Section 3.1 and Section 3.2, OT and DM cause a straight line and a random fracture, respectively. Thus, the part for a case study consists of a straight line and a curved line; the straight line and curved line are designed as OT and DM, respectively. As a result, cracks were induced in the direction desired by the user as shown in Figure 7b. In conclusion, this case study demonstrated the potential of combining OT and DM technologies to effectively control crack propagation and thereby optimize design for the purpose of protecting critical components. This approach can be further developed and applied in a variety of industries where precise crack control is essential to improve product life and sustainability.

## 4. Conclusions

This study provided insights into the design and fabrication of additively manufactured structures with controlled crack propagation characteristics. The key findings and conclusions are as follows:

1. Lattice structure type significantly influences crack propagation patterns. OT tends to exhibit straighter crack paths, while DM shows more random fracture patterns.
2. Lattice density plays a crucial role in impact energy absorption. Higher-density lattices demonstrate superior impact energy compared to lower-density structures for both OT and DM configurations.
3. PBF affects the mechanical properties of the fabricated structures. Higher laser energy generally results in improved impact energy absorption, particularly for higher-density lattices.
4. The geometry of the specimen (angle vs. stair) interacts with lattice type to influence crack propagation behavior. DM shows better crack propagation in angle specimens, while OT exhibits superior impact energy in stair-shaped specimens.
5. The combination of OT and DM can be effectively used to guide crack propagation along predetermined paths, as demonstrated in the case study. This approach offers the potential for protecting critical components.

These results contribute to the growing body of knowledge on design for additive manufacturing, particularly in the context of functional lattice structures. The ability to control crack propagation by designing lattice structures and process parameters provides a new technology for creating components with tailored mechanical properties and failure modes. Future research is needed to extend the proposed approach to other materials and AM technologies. Furthermore, computational modeling and simulation techniques can be developed to predict and optimize crack propagation behavior in complex lattice structures. The results of this study have important implications for a variety of industries where controlled fracture behavior is important, such as aerospace, automotive, and biomedical.

## Data Availability

The original contributions presented in the study are included in the article, further inquiries can be directed to the corresponding author.
